# Hydroxychloroquine-Associated Thrombotic Thrombocytopenic Purpura

**DOI:** 10.4274/tjh.galenos.2020.2020.0322

**Published:** 2020-11-19

**Authors:** Fatma Arıkan, Yasin Yıldız, Tarık Ercan, Özen Oruç, Seçkin Akçay, Fergun Yılmaz, Tayfur Toptaş, Tülin Tuğlular

**Affiliations:** 1 Marmara University Faculty of Medicine, Department of Hematology, İstanbul, Turkey; 2 Marmara University Hospital, Clinic of Internal Medicine, İstanbul, Turkey; 3 Ümraniye Training and Research Hospital, Clinic of Endocrinology, İstanbul, Turkey

**Keywords:** Hydroxychloroquine, Thrombotic thrombocytopenic purpura, Severe acute respiratory syndrome-related coronavirus

## To the Editor,

Although there have been inconsistent publications on the activity and safety of hydroxychloroquine (HQ), it is recommended by several treatment guidelines to be used for all patients with symptomatic COVID-19 disease. There were several concerns regarding the treatment-related side effects. The most important side effects include QT prolongation and visual-field defects.

A 65-year-old man with chronic obstructive pulmonary disease was admitted to the hospital with the complaints of cough and chest and back pain. Physical examination was unremarkable. Computerized tomography and angiography of the chest revealed bilateral emphysematous changes. There were no findings suggesting venous thromboembolism. Laboratory finding were as follows: hemoglobin (Hb), 14.4 g/dL; mean corpuscular volume, 96.4 fL; lymphocyte count, 3070/µL; platelet count (PLT), 150,000/µL; prothrombin time, 14 s; and international normalized ratio, 1.0. The patient had no fever or shortness of breath. There was no previous history of travel abroad or close contact with anyone who was SARS-CoV-2-positive. A nasal swab was obtained for SARS-CoV-2 polymerase chain reaction (PCR). In the outpatient setting, HQ was started without waiting for the test results.

He was reevaluated on the third day of treatment. There was no improvement in his complaints. The SARS-CoV-2 PCR test result was negative. Laboratory results at his second admission were as follows: Hb, 10.8 g/dL; PLT, 31,000/µL; lactate dehydrogenase (LDH), 1281 U/L (upper limit of normal: <248 U/L); and creatinine, 1.7 g/dL (upper limit of normal: <1.2 mg/dL). The patient had aphasia. Cranial computerized tomography was consistent with infarction of the medial cerebral artery. He was hospitalized with the suspicion of thrombotic thrombocytopenia purpura (TTP). Direct Coombs test was negative. There were 10% schistocytes in the peripheral blood smear. Disseminated intravascular coagulation was ruled out. His PLASMIC score was 6, which indicated a high probability of TTP [1].

After a blood sample was taken for ADAMTS13 analyses, HQ was ceased and exchange plasmapheresis with 1.5 volumes was started. Methylprednisolone (1 mg/kg/day) and folic acid supplementation was commenced. The ADAMTS13 level, ADAMTS13 activity, and ADAMTS13 inhibitor levels were <0.012 (0.19-0.81) IU/ml, <0.2% (40%-100%), and 90 (<12) U/mL, respectively. On the fourth day of his admission, thrombocytopenia was improved and LDH level returned to the normal range. On day 7, plasmapheresis was discontinued.

Acute immune reactions and dose-dependent toxicity play important roles in drug-related TTP etiology. The most common drug known to be related to TTP is quinine [2]. Quinine-dependent antibodies have been shown to induce TTP through immune-mediated mechanisms by interacting with platelets and other cells. HQ belongs to the 4-aminoquinoline class and is an amine acidotropic form of quinine. There are two case reports of possible HQ-related TTP in the literature. A 64-year-old woman with rheumatoid arthritis developed TTP after 3 doses of HQ [3] and a 34-year-old woman with a diagnosis of systemic lupus erythematosus had TTP under HQ treatment [4]. However, in the latter case, the relation of HQ and TTP was suspicious. Our case is the third case of possible HQ-related TTP in the literature. The adverse drug reaction probability score was calculated as 4 and adverse drug reaction was thus assigned to the “possible” category [5] (Table 1). It may be considered that TTP may be among the rare side effects in treatment with HQ.

## Figures and Tables

**Table 1:**
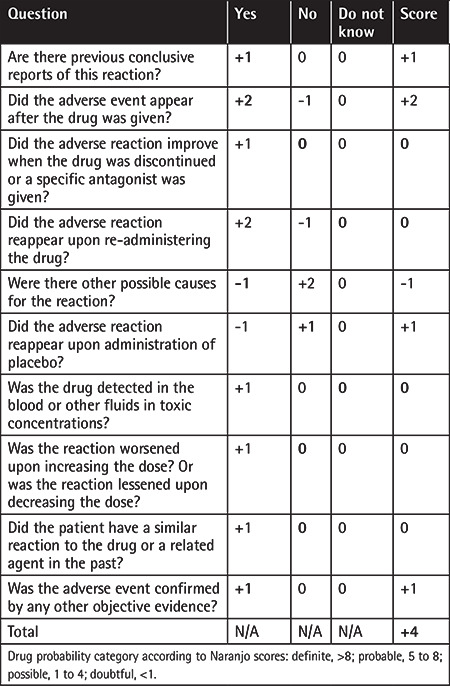
Adverse drug reaction probability scale (Naranjo Scale).

## References

[1] Bendapudi PK, Hurwitz S, Fry A, Marques MB, Waldo SW, Li A, Sun L, Upadhyay V, Hamdan A, Brunner AM, Gansner JM, Viswanathan S, Kaufman RM, Uhl L, Stowell CP, Dzik WH, Makar RS (2017). Derivation and external validation of the PLASMIC score for rapid assessment of adults with thrombotic microangiopathies: a cohort study. Lancet Haematol.

[2] George JN (2010). How I treat patients with thrombotic thrombocytopenic purpura: 2010. Blood.

[3] Fromm LM (2018). Suspected hydroxychloroquine-induced thrombotic thrombocytopaenic purpura. J Pharm Pract Res.

[4] Mar N, Mendoza Ladd A (2011). Acquired thrombotic thrombocytopenic purpura: puzzles, curiosities and conundrums. J Thromb Thrombolysis.

[5] Naranjo CA, Busto U, Sellers EM, Sandor P, Ruiz I, Roberts EA, Janecek E, Domecq C, Greenblatt DJ (1981). A method for estimating the probability of adverse drug reactions. Clin Pharmacol Ther.

